# Effect of Regional Anesthesia vs. General Anesthesia Technique on Perioperative Outcomes in Geriatric Patients With Hip Fracture: Narrative Review

**DOI:** 10.1155/anrp/8083966

**Published:** 2026-05-08

**Authors:** Hong Tao, Shuguang Yang, Wei Mei

**Affiliations:** ^1^ Department of Anesthesiology, Tongji Hospital, Tongji Medical College, Huazhong University of Science and Technology, Wuhan, 430031, Hubei, China, hust.edu.cn

**Keywords:** geriatric patients, hip fracture, perioperative outcome, regional anesthesia

## Abstract

The rising incidence of hip fractures in aging populations necessitates optimized perioperative management to mitigate complications. This narrative review evaluates the impact of regional anesthesia (RA) and analgesia techniques compared to general anesthesia (GA) on perioperative outcomes in geriatric patients undergoing hip fracture surgery. Current evidence reveals no consistent mortality benefit for RA over GA. Multicenter trials such as REGAIN and RAGA found no significant difference in 30‐ to 60‐day survival. Similarly, spinal anesthesia does not show a clear superior advantage over GA on postoperative delirium (POD) in geriatric hip fracture patients. For pneumonia and other major complications, current evidence remains inconclusive, with more well‐designed studies needed in the future. Peripheral nerve blocks (PNBs), including femoral block, fascia iliaca compartment block, and pericapsular nerve group blocks, demonstrate superior perioperative analgesia by reducing opioid consumption and early pain scores. However, evidence on PNBs’ effects on mortality or major complications remains limited. Overall, RA and GA exhibit comparable mortality outcomes, while PNBs enhance pain management but require further high‐quality trials to assess long‐term benefits. Current data highlight the need for individualized anesthesia strategies and robust RCTs to clarify optimal practices in high‐risk geriatric populations.

## 1. Introduction

The global aging population presents a growing challenge for healthcare systems. According to the WHO World Report on Aging and Health, the proportion of elderly people in Europe is projected to increase twofold by 2050 [1]. Globally, as the population is aging rapidly, the demand for hip fracture surgery is also increasing. In urban China, the absolute number of hip fractures in people aged 55 and older has increased about fourfold from 2012 to 2016, driven by population aging [2]. Each year, about 1.6 million people sustain a hip fracture worldwide, and there has been a continuous global increase in hip fracture surgeries over the last decades [3]. Successful surgery and postoperative functional recovery in the elderly remain clinically challenging. Potential serious postoperative complications, such as cognitive impairment and walking disabilities observed in elderly patients, would impair the quality of life; proactive management and prevention of these complications are therefore critical to optimize the postoperative outcomes of geriatric hip fracture surgery [4]. Patients with multiple comorbidities are widely recognized to face a high risk of death. Up to 95% of the patients with hip fractures have at least one major comorbidity upon hospital admission [5]. In clinical practice, most anesthesia practitioners tend to perform regional anesthesia (RA) more than GA for patients with delirium, pneumonia, myocardial infarction (MI), and other serious complications, which may introduce potential selection bias in related clinical research. This review will focus on the effect of RA and analgesia techniques on perioperative outcomes in elderly patients undergoing hip fracture surgery.

Randomized controlled trials (RCTs) provide the highest level of evidence for causal inference, whereas observational studies may introduce selection bias that favors apparent “benefits” of RA. Therefore, this review critically appraises both RCT and observational evidence, emphasizing the distinction between statistical significance and clinical meaningfulness.

## 2. Effects of RA vs. GA (RAGA) in Geriatric Patients With Hip Fracture

### 2.1. Neuraxial Anesthesia vs. GA

#### 2.1.1. Mortality

In recent years, growing research has investigated the influence of anesthesia type in postoperative complications, rehabilitation, and mortality in geriatric patients after hip fracture surgery, with most studies comparing the effects of intravertebral anesthesia with GA. Mortality remains the most concerning postoperative outcome. Desai et al. conducted a retrospective large‐scale study (*n* = 16,695, 2009–2014) from the Kaiser Permanente Hip Fracture Registry database. The results suggested that GA was associated with a higher risk of 90‐day mortality (HR = 1.2 [95% CI: 1.11–1.35]; *p* < 0.001) compared to RA. However, no significant difference was observed in other 90‐day postoperative outcomes, including MI, pneumonia, pulmonary embolism (PE), and deep vein thrombosis (DVT) [6]. Ahn et al. performed a nationwide population‐based retrospective study to assess the effects of the anesthesia method on mortality and delirium in geriatric patients undergoing surgery for hip fractures through the Korean National Health Information Database (*n* = 96,289, 2009–2015). After prior propensity score matching (PSM), the GA group showed slightly higher overall 30‐day mortality compared to the RA group (2.55% vs. 2.24%, *p* = 0.0047). While before PSM, both groups showed the same overall 30‐day mortality rate as 2.45% [7]. Similarly, Rodkey et al. performed another retrospective cohort study analyzing the data from the American College of Surgeons National Surgical Quality Improvement Program (ACSNSQIP) database (*n* = 23,649, 2015–2018) with PSM treatment and found no significant difference in 30‐day mortality between the GA cohort and the spinal anesthesia cohort (4.3% vs. 4.1%, *p* = 0.054) [8]. One larger sample‐size retrospective cohort study (*n* = 60,897 ACSNSQIP, 2015–2016) also concluded that anesthesia type was not associated with 30‐day all‐cause postoperative mortality in elderly patients undergoing partial hip arthroplasty (OR = 0.85, 95% CI: 0.59–1.22) or total hip arthroplasty (OR = 0.68, 95% CI: 0.43–1.08), though PSM was not applied in this study [9]. But, another large‐size retrospective cohort study (*n* = 40,527, ACSNSQIP, 2016–2019) demonstrated that spinal anesthesia was linked to reduced postoperative morbidity and mortality in hip fracture surgery patients when compared to GA after PSM treatment [10]. Nishi et al. conducted a retrospective cohort study to examine the effect of anesthesia type on 30‐day or 90‐day mortality and perioperative length of stay (LOS) in elderly patients who underwent hip fracture surgery using the Fukuoka Prefecture’s Claims database (*n* = 16,125, 2012–2016). In this study, no significant difference was observed in 30‐day and 90‐day mortality between GA group and RA group both before (*p* = 0.411, *p* = 0.543) and after PSM (*p* = 0.498, *p* = 0.594) [11] The Regional versus GA for Promoting Independence after Hip Fracture (REGAIN) trial, a multicenter randomized superiority trial (*n* = 1600, 2018–2021), compared the effect of anesthesia type in 60‐day survival and ambulation recovery after hip fracture surgery. The results indicated no superiority for spinal anesthesia in elderly patients (mean age 78 years) compared to GA (18.5% vs. 18%, RR = 1.03 [95% CI: 0.84–1.27]). Notably, approximately 15% patients randomized to receive spinal anesthesia ultimately crossed over to GA, which potentially diminished this study’s statistical power, although the primary outcomes remained consistent in an instrumental variable analysis that accounted for nonadherence to treatment assignment [12]. The RAGA trial, another randomized multicenter clinical trial (*n* = 941, mean age 76.5 years) indicated that the all‐cause 30‐day mortality as secondary outcome also did not significantly differ between RA and GA (1.7% vs. 0.9%; unadjusted RD = −0.8% [95% CI: −2.2%–0.7%]; unadjusted RR = 2.0 [95% CI: 0.6–6.5]) [13]. Furthermore, the recent long‐term outcomes study of the REGAIN trial found that anesthesia types did not impact survival at up to 1 year after hip surgery (HR = 1.08 [95% CI: 0.81–1.44]; *p* = 0.59) [14]. Upon the results of large multicenter RCTs and recent years’ retrospective studies from population databases, RA does not show a clear superior advantage over GA in terms of mortality in geriatric patients after hip fracture surgery.

However, several important methodological considerations should be acknowledged when interpreting these findings. First, the majority of cited database studies employ PSM, which cannot adjust for unmeasured confounders such as frailty, baseline cognitive function, prefracture mobility, or nutritional status, factors strongly associated with both anesthetic choice and outcomes. The apparent “benefit” of RA in these observational studies is highly susceptible to selection bias, as clinicians may preferentially select healthier patients for neuraxial techniques while reserving GA for those with contraindications to regional techniques or more severe comorbidities. Therefore, these associations may reflect residual confounding rather than causal effects. Second, although the REGAIN trial and RAGA trial stated no significant difference, respectively, in 60‐day survival and 30‐day mortality between spinal and GA, Restrepo et al. evaluated the fragility of the two trials and found that the number of patients need to change the significance was lesser than the numbers of patients lost to follow‐up, which suggested that these findings should be interpreted with appropriate caution [15]. A summary of major studies in terms of postoperative mortality comparing RA and GA for hip fracture surgery is presented in Table 1.

**TABLE 1:** Summary of major studies comparing regional anesthesia and general anesthesia for hip fracture surgery.

Study (Year)	Study type	Sample size	Primary outcome	Key finding	Critical appraisal
Ahn et al. [7] (2019)	PSM cohort	96,289	30‐Day mortality	2.55% vs 2.24% (*p* = 0.0047)	Statistically significant; residual confounding likely
Rodkey et al. [8] (2022)	PSM cohort	23,649	30‐Day mortality	4.3% vs 4.1% (*p* = 0.054)	Not statistically significant; borderline result
Chu et al. [16] (2015)	PSM cohort	182,307	In‐hospital mortality	OR 1.24 (*p* < 0.001)	Large ORs for rare outcomes; absolute differences small
Neuman et al. [12] (2021)	RCT	1600	60‐Day survival	18.5% vs 18% (RR = 1.03)	No significant difference; ∼15% crossover
Li et al. [13] (2022)	RCT	950	Postoperative delirium	20.5% vs 19.7% (*p* = 0.79)	No significant difference; low fragility index
Vail et al. [14] (2024)	RCT follow‐up	1600	1‐Year survival	HR = 1.08 (*p* = 0.59)	Long‐term outcomes similar

Abbreviations: HR, hazard ratio; OR, odds ratio; PSM, propensity score matching; RCT, randomized controlled trial; RR, relative risk.

#### 2.1.2. Delirium

Delirium is a frequent complication of hip fractures in older adults, linked to higher rates of cognitive decline, institutionalization, and mortality [17]. Careful selection of the anesthesia method for hip fracture surgery is recommended to reduce the incidence of postoperative delirium (POD). Cognitive impairments after surgery present as two types: POD and postoperative cognitive dysfunction (POCD). POD is marked by fluctuating levels of alertness, overall cognitive function, and memory, potentially following a circadian rhythm. POCD signifies a reduction in cognitive performance from the initial level that arises after surgery and might either recover or remain for an unspecified time. Patients who received joint replacement surgery faced a 7%–75% risk of developing cognitive decline and delirium postsurgery, influenced by the definition, patient demographics, timing of assessment, and measurement tools [18]. Uzoigwe et al. conducted a retrospective study through the National Hip Fracture Database of the United Kingdom (*n* = 1224, mean age 81.5 years, 2016–2018) to determine the risk factors associated with POD and POCD, using the assessment tool of Abbreviated Mental Test Score (AMTS). The results indicated that AMTS was a predictor of POD with AMTS of 8 as a threshold, yielding 81% specificity (95% CI: 78%–83%) and 92% sensitivity (95% CI: 88%–95%). Multivariate logistic regression identified ASA grade as the only predictor for POCD (OR = 2.6 [95% CI: 1.7–4.0]; *p* < 0.001), while no significant association was observed between age, sex, mode of anesthesia, use of cement, and POCD [19]. Recently, Song et al. performed a prospective cohort study (*n* = 138) to investigate the effect of anesthesia type on circadian melatonin rhythm and POD in elderly patients undergoing hip fracture surgery. They found that patients in the GA group experienced more circadian rhythm disruption, more awakenings, more sleep deprivation, poorer sleep quality on surgery day, and a higher incidence of POD than patients in the spinal anesthesia group (34.8% vs. 17.4%, *p* = 0.020) [20]. A large sample‐size retrospective study (*n* = 18,754, age ≥ 60 years) from the NSQIP database (2016–2017) suggested preoperative dementia and POD independently correlated with 30‐day mortality, and the mortality odds further increased when preoperative dementia and POD were both present (OR = 2.25, *p* < 0.001) [21]. The RAGA trial investigated the effect of anesthesia type on incidence of POD as primary outcome in elderly patients after hip fracture surgery and found that RA without sedation was not associated with a significant reduction in the incidence of POD during the first seven postoperative days when compared to GA (6.2% vs. 5.1%, unadjusted RD = 1.1 [95% CI: −1.7%–3.8%]; *p* = 0.48 and unadjusted RR = 1.2 [95% CI: 0.7–2.0]; *p* = 0.57) [13]. Similarly, the REGAIN trial, which investigated the incidence of POD as a secondary outcome, found also no difference between the two anesthesia types (20.5% vs. 19.7%, RR = 1.04 [95% CI: 0.84–1.30]) [12]. The RAGA trial authors suggested that the lower POD incidence observed might be due to the use of a diagnostic tool for delirium, the short confusion accessing method (CAM), which was recommended by the National Institute for Health and Care Excellence; the higher proportion of patients with ASA I and II status; and the 23.2% use of peripheral nerve block (PNB) in GA group [14, 22]. The 12‐month follow‐up study in patients from the RAGA trial explored the incidence of 12‐month postoperative cognitive decline and found no significant difference between patients receiving general or RA for hip fracture surgery (29.7% vs. 25.4%; unadjusted OR = 1.2 [95% CI: 0.7–2.1]; *p* = 0.43) [23]. But, Restrepo et al. evaluated the vulnerability of the existing evidence supporting the debate on anesthesia types for hip fracture surgery, including the REGAIN and RAGA trials, and showed that fragility index (FI) and reverse FI (rFI) in these two trials were generally low when considered alone or lower than the number of patients who were lost to follow‐up, which mean the current evidence could be considered fragile [15]. However, with POD rates around 20%, the reclassification of just 3–5 patients could render the *p* value nonsignificant, indicating that the absence of a statistically significant difference does not rule out a small but potentially important clinical effect. Thus, upon the current evidence, spinal anesthesia does not show a clear superior advantage over GA on POD in geriatric patients undergoing hip fracture surgery; more RCTs with large sample sizes and high quality in the high‐risk elderly population are still needed to draw a clear conclusion.

#### 2.1.3. Pneumonia and Other Complications

Chu et al. performed a retrospective study using data from China Taiwan’s in‐patient claims database (*n* = 182,307, 1997–2011) to evaluate the impact of anesthesia type on in‐hospital outcomes. After PSM, the results demonstrated that regardless of hip fracture type (intracapsular or extracapsular), GA was consistently related to increased risk of in‐hospital mortality (OR = 1.24 [95% CI: 1.15–1.35]; *p* < 0.001), respiratory failure (OR = 2.71 [95% CI = 2.38–3.08]; *p* < 0.001), ICU admission (OR = 1.95 [ 95% CI: 1.87–2.07]; *p* < 0.001), and need for ventilator support (OR = 6.08 [95% CI: 5.59–6.01]; *p* < 0.001) than neuraxial anesthesia [16]. Similarly, another large PSM‐treated retrospective study (*n* = 23,694, NSQIP database, 2015–2018) revealed spinal anesthesia was associated with less DVT and less overall complications, though it had an increased risk of urinary tract infection compared to GA [8]. However, several studies concluded the other way. Desai et al. found no significant difference in 90‐day postoperative outcomes, such as PE, pneumonia, MI, and DVT, between different anesthesia types in their retrospective study [6]. A PSM‐treated cohort study of elderly patients undergoing hip fracture surgery (*n* = 19,809 Japanese Diagnosis Procedure Combination administrative claims database, 2010–2019) reported no significant difference in incidence of aspiration pneumonia (1.8% vs. 1.5%, *p* = 0.158) and other pulmonary complications (1.5% vs. 1.5%, *p* = 0.893), such as pneumonia, atelectasis, pneumothorax, bronchitis, pulmonary edema, and PE, between general and spinal anesthesia groups [24]. Another PSM‐treated Chinese single‐center retrospective cohort study (*n* = 3147, 2016–2020) conducted in patients undergoing hip fracture surgery identified the incidence and risk factors of postoperative pneumonia, including age, male gender, respiratory disease, heart disease, cerebrovascular disease, liver disease, preoperative stay, and GA, with a cumulative incidence of 5.8% [25].

When evaluating complications data from observational studies, several statistical considerations merit attention. First, studies that examine multiple endpoints without adjustment for multiplicity inflate the risk of false‐positive findings. Second, the clinical significance of reported differences must be assessed alongside statistical significance. For example, Chu et al. reported substantial odds ratios for respiratory failure (OR = 2.71) and ventilator support (OR = 6.08) with GA [16], but the baseline incidence of these outcomes is low, resulting in small absolute risk differences. Clinicians should consider both the relative and absolute effects when weighing the risks and benefits of anesthetic techniques. Third, the inconsistent findings across studies likely reflect heterogeneity in patient populations, outcome definitions, and anesthesia protocols. The lack of standardized definitions for complications such as pneumonia and respiratory failure across administrative databases limits the comparability of results. In addition, temporal changes in perioperative care practices during the study periods may also confound the observed associations. Notably, the REGAIN and RAGA trials demonstrated comparable occurrence rates of major postoperative complications between spinal and GA, including POD, LOS, and pneumonia [14, 26]. Current evidence remains inconclusive regarding anesthesia types’ impact on postoperative pneumonia and other major complications; more well‐defined studies are guaranteed.

### 2.2. PNB vs. GA

The continuous lumbar plexus block (CLSB) has been employed as a peripheral anesthesia technique in hip fracture surgery for decades, demonstrating comparable efficacy to neuraxial anesthesia [27]. However, its routine use remains limited due to its technical demands and for being time‐consuming. Application of ultrasound guidance expanded CLSB’s utility in patients with anatomy challenges or concomitant anticoagulant/antiplatelet therapies [26]. Thus, CLSB should be considered as a viable alternative when RA is strongly preferred, but neuraxial anesthesia is contraindicated. A single‐center retrospective study in elderly patients with hip fractures (*n* = 70, ASA III‐IV, 2010–2012) reported effective perioperative pain control using CLSB without major anesthesia‐related complications [28]. Another PSM‐adjusted single‐center retrospective study of patients undergoing primary total hip arthroplasty (*n* = 902, age ≥ 60 years) revealed a significant reduction in delirium incidence comparing PNB to GA (2.3% vs. 9.0%, respectively; RR = 0.233 [95% CI: 0.064–0.845]; *p* = 0.030) [29]. Häusler performed a single‐center cohort study of elderly patients (*n* = 407, mean age 85.2 years) with hip fracture and found significantly lower postoperative opioid consumption in the femoral nerve block (FNB) group compared to the continuous femoral nerve (FN) catheter group and the GA group at the time point of recovery room and 48 h after surgery, though no significant difference was observed regarding POD and mortality between groups [30]. Interestingly, one recent PSM‐adjusted retrospective cohort study of patients undergoing unilateral hip arthroplasty (*n* = 316, aged ≥ 65 years, 2014–2019) reported that PNB (CLSB with L1 paravertebral block) showed significantly lower 30‐day (2.2% vs. 10.1%, *p* = 0.029) and 90‐day (3.4% vs. 12.4%, *p* = 0.026) mortality compared to spinal anesthesia [31]. In general, studies exploring the effects of PNB as a sole anesthesia technique on postoperative outcomes in patients undergoing hip fracture surgery remain limited; further investigations are required to establish a conclusion on this issue.

Previous studies have reported that most patients with hip fractures did not receive adequate pain relief [32]. However, adequate pain management is important since severe pain may cause mental disorders and delirium [33]. PNB technique, including FNB, fascia iliaca compartment block (FICB), and pericapsular nerve group (PENG) block, has been employed for pain relief in patients who received surgery for hip fractures under GA.

#### 2.2.1. FNB

Uysal et al. conducted an RCT comparing the effects of FNB and paracetamol on preoperative pain control and POD incidence in geriatric patients (*n* = 110, mean age more than 80 years) undergoing spinal anesthesia for trochanteric femur fracture within 48 h. The patients in the FNB group had significantly lower visual analog scale (VAS) scores at the 4^th^ hour after the first preoperative treatment (3.32 ± 0.92 vs. 4.47 ± 1.06, *p* < 0.01) and during spinal anesthesia positioning (4.02 ± 1.80 vs. 5.55 ± 1.77, *p* < 0.01) than patients in the paracetamol group [34]. In contrast, another single blind RCT (*n* = 73, 2015–2017) found no significant difference in postoperative mean pain score (up to 5 days) and perioperative opioid use between the preoperative FNB group and the standard medication group [35].

#### 2.2.2. Fascial Iliaca Compartment Block

Multiple prospective studies supported the opioid‐sparing effect of FICB in patients with hip fractures. Garlich et al. reported reduced preoperative opioid consumption in two prospective observational studies. One prospective observational study (*n* = 725, mean age of 84.2 years) suggested that preoperative FICB reduce preoperative opioid consumption but not postoperative opioid consumption [36]. The other prospective cohort study (*n* = 102, mean age of 83.3 years) also suggested that faster preoperative FICB bring less opioid consumption [37]. An RCT in geriatric patients with hip fractures showed preoperative FICB significantly reduced postoperative opioid consumption for severe pain (0.4 vs. 19.4 mg, *p* = 0.05) and increased patient‐reported satisfaction by 31% compared to standard medication [38]. Continuous FICB significantly reduced postoperative pain score and opioid consumption during first 3 days after hip fracture surgery compared to medication, though no significant difference was observed between single shot FICB and continuous FICB within 2 days after hip surgery for fractures [39, 40] A meta‐analysis with trial sequential analysis confirmed FICB’s efficacy in reducing pain scores and opioids requirement of geriatric patients with hip fracture in emergence department [41]. The Pain Threshold Index (PTI) reflects the nociceptive state in unconscious patients and could replace the numerical rating scale in surgical patients under GA. An RCT (*n* = 87) reported that preoperative combined nerve block (FICB combined with sacral plexus block and superior cluneal nerve block) significantly decreased PTI in patients with hip fractures compared to GA [42]. Regarding postoperative complications, Hao et al. conducted an RCT (*n* = 85) in elderly patients with hip fracture and revealed that continuous FICB significantly reduced preoperative VAS scores and POD incidence (13.9% vs. 35.7%, *p* = 0.018) compared to the control group [43].

#### 2.2.3. PENG Block

Short et al. reported that the anterior hip capsule is predominantly innervated by the FN, obturator nerve (ON), and accessory ON (AON). Of which, AON and FN play a predominant role in anterior hip innervations compared to previous studies. The major articular branches from the FN and AON are reliably found between the anterior inferior iliac spine and the iliopubic eminence, while the ON is situated near the inferomedial acetabulum [44]. This updated anatomical knowledge facilitated the development of PENG, an interfacial plane block technique designed to provide analgesia to the hip joint [45]. Natrajan et al. conducted a double‐blind RCT (*n* = 24) to assess the postoperative analgesic effect of FICB and PENG in patients undergoing surgery for hip fractures using numerical rating scores (NRSs). The results reported significantly lower NRS at 1^st^ (*p* = 0.035) hour and 4^th^ hour (*p* = 0.001) in postanesthesia care unit (PACU) in the PENG group than in the FICB group, and no significant difference was observed at other time points up to postoperative 24 h between the two groups [46]. Similarly, Hua et al. performed another RCT (*n* = 48) comparing the analgesic effect of PENG and FICB in geriatric patients with femoral neck fracture using VAS scores. The results demonstrated significantly lower VAS scores in the PENG group than in the FICB group at the interval between block completion and spinal anesthesia [47]. One recent Bayesian network meta‐analysis analyzing 63 RCTs evaluated the comparative efficacy of PNBs in preoperative pain management for hip fractures and concluded that the PENG block provides better pain relief at 2 h after block placement compared to FNB and FICB. However, the evidence level of this study was rated as low to very low due to limited confidence in the outcome, and no significant difference was observed in length of hospital stay (LOS) among the different block groups [48]. A Cochrane database systematic review analyzed 49 trials comparing PNBs use (preoperative analgesia, postoperative analgesia, and adjunctive to GA) versus control (no or sham block) in patients with hip fractures, which concluded that PNBs reduce movement‐related pain within 30 min after block and reduce the risk of acute confusional state with high certainty evidence. The review emphasized high‐quality trials with proper sample sizes to investigate the influence of PNBs on MI and mortality [49].

Ultrasound‐guided PNBs (FNB, FICB, and PENG block) demonstrate consistent opioid‐sparing effects and improved pain control in the immediate perioperative period. However, evidence regarding their impact on mortality, major complications, and long‐term functional outcomes remains limited. These techniques should be considered as components of multimodal analgesic pathways rather than standalone interventions to improve survival.

## 3. Conclusions and Perspectives

In sum, current evidence suggests that neither neuraxial nor GA confers an independent survival advantage in older patients with hip fracture [12, 13, 50]. Ultrasound‐guided PNBs improve perioperative pain management and opioid exposure and are often successfully applied in high‐risk populations, particularly patients with severe cardiopulmonary comorbidities [51, 52]. However, PNBs’ impact on other major postoperative outcomes remains underpowered. This may underscore that any meaningful improvement in outcome demands a paradigm shift from mere technique selection to a perioperative multimodal intervention framework. In conclusion, the choice of the anesthetic technique should be individualized based on patient characteristics, comorbidities, preferences, and institutional expertise. GA remains a safe and appropriate option for many patients, and the available evidence does not support a universal recommendation for RA to improve survival. Several priorities for future research emerge from this review. First, adequately powered randomized trials examining patient‐centered outcomes such as functional recovery, quality of life, and long‐term cognitive function are needed. Second, the development and validation of frailty‐adjusted outcome measures would improve risk stratification and allow for more nuanced interpretation of results. Third, studies examining the optimal integration of PNBs within enhanced recovery pathways could inform clinical practice.

## Author Contributions

All authors listed have significantly contributed to the development and writing of this article.

## Funding

This research did not receive any specific grant from funding agencies in the public, commercial, or not‐for‐profit sectors.

## Conflicts of Interest

The authors declare no conflicts of interest.

## Data Availability

No dataset was generated or analyzed in the article.
